# Different tau species lead to heterogeneous tau pathology propagation and misfolding

**DOI:** 10.1186/s40478-018-0637-7

**Published:** 2018-11-29

**Authors:** Simon Dujardin, Séverine Bégard, Raphaëlle Caillierez, Cédrick Lachaud, Sébastien Carrier, Sarah Lieger, Jose A. Gonzalez, Vincent Deramecourt, Nicole Déglon, Claude-Alain Maurage, Matthew P. Frosch, Bradley T. Hyman, Morvane Colin, Luc Buée

**Affiliations:** 1 Univ. Lille, Inserm, CHU-Lille, UMR-S 1172, Alzheimer & Tauopathies, School of Medicine, 1 rue Polonovski, 59045 Lille, France; 2 Department of Neurology, Massachusetts General Hospital, MassGeneral Institute of Neurodegenerative Diseases (MIND), Charlestown, MA USA; 3 Department of Neurology, Harvard Medical School, Boston, MA USA; 4 Lausanne University Hospital (CHUV), Neuroscience Research Center (CRN), Laboratory of Neurotherapies and Neuromodulation (LNTM), CH-1011 Lausanne, Switzerland

**Keywords:** Tau, Propagation, Isoforms, Misfolding, Alzheimer’s disease, Heterogeneity

## Abstract

Tauopathies are a heterogeneous group of pathologies characterized by tau aggregation inside neurons. Most of them are sporadic but certain tauopathies rely on tau gene (MAPT) mutations. They particularly differ from one to another by their different neuropathological signatures e.g. lesion shapes, regions affected and molecular composition of aggregates. Six isoforms of tau exist, but they do not all co-aggregate in each tauopathy but rather have a unique signature for each one. In some tauopathies such as Alzheimer’s disease (AD), tau protein aggregation follows stereotypical anatomical stages. Recent data suggest that this progression is due to an active process of tau protein propagation from neuron-to-neuron. We wondered how tau isoforms or mutations could influence the process of tau aggregation and tau propagation. In human neuropathological material, we found that MAPT mutations induce a faster misfolding compared to tau found in sporadic AD patients. In the rat brain, we observed cell-to-cell transfer of non-pathological tau species irrespective of the tested isoform or presence of a mutation. By contrast, we found that the species of tau impact the propagation of tau pathology markers such as hyperphosphorylation and misfolding. Indeed, misfolding and hyperphosphorylated tau proteins do not spread at the same rate when tau is mutated, or the isoform composition is modified. These results clearly argue for the existence of specific folding properties of tau depending on isoforms or mutations impacting the behavior of pathological tau species.

**Electronic supplementary material:**

The online version of this article (10.1186/s40478-018-0637-7) contains supplementary material, which is available to authorized users.

## Introduction

Tau is a microtubule-associated protein mainly found in the axonal part of neurons. Resulting from an alternative splicing mechanism, six major isoforms of tau coexist in the human brain with the presence of either 3 or 4 repeated sequences (named below as 3R-tau or 4R-tau) known as the microtubule-binding regions [8, 28]. The hyperphosphorylation and deposition of tau proteins in insoluble aggregates inside neurons are a hallmark of around 20 pathologies called tauopathies including the well-known Alzheimer’s disease (AD) [8, 25]. These aggregates progressively invade the whole neuron and form specific intraneuronal lesions named neurofibrillary degeneration (NFD) ultimately leading to cell death. However, the kinetics of this pathological cascade as well as the exact factors leading to cell death are still poorly understood [61]. Furthermore, even if tauopathies share common features, this group of pathologies is very heterogeneous with a vast variety of clinical presentations including fronto-temporal dementias (e.g. Pick’s disease (PiD)), movement disorders/parkinsonism (e.g. progressive supranuclear palsy (PSP)) or Alzheimer’s type dementia such as AD or argyrophilic grain disease (AGD) [39]. This heterogeneity may be explained by strong histopathologic differences and differential laminar and regional brain distribution but also by molecular variations such as isoform composition and posttranslational modifications. For example, in AD, the six isoforms of tau co-aggregate, in contrast to pathologies like PSP or AGD, in which only the 4R-tau isoforms aggregate, and Pick’s disease, where the aggregates are only comprised of 3R-tau [39]. Adding additional complexity, around 50 autosomal dominant mutations in the tau gene (MAPT) have been reported to promote strong tau aggregation and clinically lead to dramatic fronto-temporal lobar degeneration (formerly FTDP-17 now referred to as genetic FTLD-Tau) [21, 23].

Likely due to the molecular heterogeneity of tauopathies, different morphologies of lesions can be observed, with mainly flame-shaped neurofibrillary tangles in AD, argyrophilic grains and/or glial lesions in AGD or PSP and Pick bodies in Pick’s disease [39]. These lesions affect different part of the brain and the pathology evolves differently. Thus, histopathological studies in some sporadic tauopathies such as AD [6, 15, 24], PSP [66, 69] and AGD [55] show that, specifically for each disease, tau lesions appear progressively and hierarchically in the brain along anatomical connections. The mechanisms underlying such evolution had remained unexplained for many years and are still poorly understood [60]. Increasing evidence both in vitro and in vivo, support the ideas that the evolution across brain areas is the result of the active propagation of NFD within the brain. Indeed, our group and others recently showed that tau assemblies are transferred from cell-to-cell and, by being taken up by a second cell, seed the aggregation of endogenous tau leading to the propagation of tau lesions in the brain [13, 14, 19, 57, 65] reviewed in [48].

Interestingly, in these studies, 4R-tau human constructs were always used to observe tau propagation/seeding. In human tauopathies, the spatio-temporal evolution of NFD was also only reported in three sporadic tauopathies in which the 4R-tau isoforms aggregate (AD, AGD and PSP). Conversely, it is still controversial whether 3R-tau can propagate in genetic FTLD-Tau (mutant tau) or Pick’s disease (3R-tau) [33].

Phosphorylation plays essential roles in tau physiology particularly by controlling its binding with microtubules [5, 49]. In AD, tau hyperphosphorylation leads to the misfolding of tau protein and its oligomerization in highly structured, insoluble aggregates [3]. Nowadays, this hypothesis has been widened to all human tauopathies. Tau misfolding is thought to be responsible for the seeding propensity of tau that ultimately becomes aggregated and insoluble [45]. This sequence of event is however not yet completely clear and some intriguing data obtained with a transgenic mouse model overexpressing mutant tau strongly suggest that the appearance of the epitopes of misfolding (particularly with the antibodies Alz50/MC1) precede hyperphosphorylation (particularly with the antibody AT8) [29]. Interestingly, in genetic FTLD-Tau (mutant tau), tau proteins show conformational changes even without hyperphosphorylation [35, 46, 67, 68]. In the present study, we re-explore these issues in human neuropathological samples and experimentally in a rat model, to understand how isoforms and mutations influence tau propensity to misfold and propagate from neuron-to-neuron.

We analyzed NFD by immunohistochemistry in different brains areas from genetic FTLD-Tau (3 different mutations) and AD patients (at different Braak stages) using either conformation-dependent or phospho-dependent antibodies. Conformational changes might occur before hyperphosphorylation only in genetic FTLD-Tau patients and not in AD. To further explore these observations, we used a rat model of tauopathies [10, 19], to examine the pathophysiological propagation of tau using different species, 3R or 4R, mutant or wild-type (WT). As previously described, 4R-tau propagates physiologically and pathologically from neuron-to-neuron to distant brain areas [19]. Interestingly, when mutant or 3R-tau constructs are used, the transfer of non-pathological species of tau remains functional but tau pathology does not spread and stays in the vicinity of the initiation site. Early conformational changes as indicated by the MC-1 immunoreactivity might facilitate aggregation and neurodegeneration rather than propagation. Therefore, our results suggest that different tau species may encounter different misfolding processes that could explain such differences.

## Materials & methods

### Viral vectors

The procedures to produce the lentiviral vectors (LVs) batches have been previously described [10, 16, 31]. Briefly, the packaging construct pCMVΔR8.92, the pRSV-Rev, pMD.2G and the human Tau cDNA (htau1N4R, htau1N4R-P301L, htau1N4R-P332S, htau1N3R or htau1N3R-P332S) were co-transfected in human 293T cells and lentiviral vectors particles were concentrated from successive ultracentrifugation of the culture medium 48 h later.

### Animals

Wistar male rats were purchased from Janvier Laboratories and housed in a temperature-controlled room maintained on a 12 h day/night cycle with food and water provided ad libitum. Animal experiments were performed in compliance with and with the approval of the local ethics committee (agreement 2015101320441671, 2016–2021), standards for the care and use of laboratory animals, and the French and European Community guidelines as well as the Massachusetts General Hospital’s Institutional Animal Care and Use Committee.

### Stereotaxic injections, cerebro-spinal (CSF) and interstitial fluids (ISF) sampling and sacrifice procedures

Rats were housed and stereotactically injected with PBS or lentiviral vectors (LVs) (Coordinates from bregma: Anteroposterior − 5.3 mm, Mediolateral +/− 6.2 mm, Dorsoventral − 7 and -6 mm; for detailed procedures see [19]) encoding either htau1N4R (*n* = 9 for histology, *n* = 3 for RNA extraction), htau1N4R-P301L (*n* = 5 for histology, n = 3 for RNA extraction), htau1N4R-P332S (*n* = 6 for histology, *n* = 3 for RNA extraction), htau1N3R (*n* = 8 for histology, n = 3 for RNA extraction) or htau1N3R-P332S (n = 5 for histology, n = 3 for RNA extraction). The negative control consists in injection of the vehicle (PBS, 1% *v*/*w* BSA; n = 3 for histology, n = 3 for RNA extraction). For histology analysis, eight months post-injection, animals were deeply anesthetized (pentobarbital 50 mg/kg) and transcardially perfused first with cold 0.9% NaCl followed by cold 4% PFA for 20 min. The brains were immediately removed, fixed overnight in 4% PFA, placed in 20% sucrose for 24 h and frozen until further use. Free-floating coronal cryostat sections (40 μm thickness) were used for immunohistochemical analysis. For RNA extraction, 2 weeks post-injection, were deeply anesthetised (pentobarbital 50 mg/kg). Brains were dissected, and 1-mm-thick coronal sections were generated using an acrylic rat brain matrix (Electron Microscopy Sciences). The sections were immediately frozen on dry ice and stored at − 80 °C until further use.

### Immunohistochemistry

For rats, immunohistochemistry procedures were previously described [19]. Briefly, sections from the entire brain were washed in PBS-0.2% Triton X-100 and treated for 30 min with H_2_O_2_ (0.3%). Non-specific binding was blocked using goat serum (1:100 in PBS, Vector) for 60 min. Sections were incubated overnight at 4 °C with the monoclonal antibodies AT8 (Thermo Scientific; MN1020–1:400; phosphorylated residues 202, 205 and 208 of tau) [42], ADx-215 [10, 54] (1:10,000; human specific total tau) or MC1/Alz50 (kind gifts from Dr. Peter Davies – 1:10,000; misfolded tau) in PBS-0.2% Triton X-100. After several washes, labelling was amplified by incubation with an anti-mouse biotinylated IgG (1:400 in PBS-0.2% Triton X-100, Vector) for 60 min followed by the application of the ABC kit (1:400 in PBS, Vector) prior to visualization with 0.5 mg/ml DAB (Vector) in Tris-HCl 50 mmol/L, pH 7.6, containing 0.075% H_2_O_2_. Brain sections were counter-stained in a cresyl violet solution (0.5%) and mounted with Vectamount (Vector) for microscopic analysis.

For human sections, 9 μm thick paraffin-embedded sections of hippocampus, temporal cortex and visual cortex of 10 human cases (Table 1) were cut using a microtome and placed on glass slides. Slides were incubated at 55 °C for 4 h before being immerged in successive 8 min baths of xylene twice, EtOH 100% twice, EtOH 95%, EtOH 70%, EtOH 50% and PBS three times. Slides were then incubated in boiling citrate buffer (citric acid anhydrous 10 mM, Tween20 0.05%, pH = 6) in a microwave at low power for 20 min. Slides were immerged in Tris-Buffered Saline (TBS) with 0.5% triton X-100 for 30 min followed by blocking with TBS, 10% Normal Goat Serum for 1 h. Slides were incubated overnight at 4 °C with primary antibodies (Alz50, kind gift of Dr. Peter Davis: 1/50 and AT8 1/400) in TBS, 5% NGS, 0.05% Triton X-100. Slides were washed 4 times with TBS and then incubated with secondary antibodies (anti-mouse IgM 568 and anti-mouse IgG 488 1/400, Invitrogen) diluted in TBS, 5% NGS. Slides were washed 4 times with TBS and counterstained with Sudan black (0.1% in 70% EtOH, filtered) for 20 min. Slides were washed 4 times with TBS and coversliped with Fluoromount G with Dapi (Thermo Fisher Scientific). Slides were scanned using an Olympus VS-120 slide scanner and then 100% of neurons were counted using the cellSens software. All human tissues come from the Lille Neurobank and the Massachusetts Alzheimer’s Disease Research center and written consent forms have been obtained accordingly to the local legislations and ethical committees. Human brains extracts were obtained from the Massachusetts Alzheimer’s Disease Research Center (grant number P50 AG005134, under IRB protocol 1999P003693) and the Lille Neurobank (CRB/CIC1403 Biobank, BB-0033-00030, agreement DC-2008-642) fulfilling criteria of the local laws and regulations on biological resources with donor consent, data protection and ethical committee review.

**Table 1:** Human case demographics

Case	Age at death	Sex (M/F)	PMI (hours)	Neuropathology diagnosis	Braak stage (if applicable)	MAPT mutation (if applicable)	Neurobank
1	70	M	12	genetic FTLD-Tau	N/A	P301L	Massachusetts ADRC
2	56	M	6	genetic FTLD-Tau	N/A	P301L	Massachusetts ADRC
3	85	F	20	genetic FTLD-Tau	N/A	P332S	Lille Neurobank
4	33	M	33	genetic FTLD-Tau	N/A	G389R	Massachusetts ADRC
5	> 90	F	8	Control	I	N/A	Massachusetts ADRC
6	68	M	27	Control	I	N/A	Massachusetts ADRC
7	63	M	16	AD	IV	N/A	Massachusetts ADRC
8	69	M	6	AD	IV	N/A	Massachusetts ADRC
9	68	M	14	AD	VI	N/A	Massachusetts ADRC
10	69	F	4	AD	VI	N/A	Massachusetts ADRC

*M* Male, *F* Female, *PMI* Post Mortem interval, *genetic FTLD-Tau* genetic FrontoTemporal Lobar Dementia-Tau, *AD* Alzheimer’s Disease, *N/A* Non-Applicable

### RNA extraction from brain sections and RT-PCR/PCR

The brain slices were lysed, and total RNA was extracted using the RNeasy Lipid Tissue kit (Qiagen) according to the manufacturer’s instructions and adjusted to 1 μg/mL. RNA were retro-transcripted using the ‘High-capacity’ cDNA reverse transcription kit (Applied Biosystems); cDNA was generated after reverse transcription of 1 μg of RNA with 4 mM of dNTPs, 10X random primers, 1 units/ μl of RNAse inhibitor, 1.25 ng/μl of oligo dT, 2.5 units/μl of MultiScribe reverse transcriptase. Real time PCR was then performed using the ‘Taqman gene expression Master Mix’ (Thermofisher) containing probe against MAPT (ref probe: Hs00902194_m1). Results were normalized to 18S transcripts (ref probe: 4308329). Reactions and data analysis were carried out with a StepOnePlus thermocycler (Applied Biosystems).

### Statistical assays

The *P* values have been determined using one-way ANOVA tests and a Tukey post-hoc test or a Pearson’s Chi-squared test with Yates’ continuity correction as indicated in the figure legend. Differences were considered to be statistically significant if *p* < 0.05.

## Results

### Differential misfolding/hyperphosphorylation profile in human MAPT mutant carriers compared to sporadic AD

**Fig. 1:**
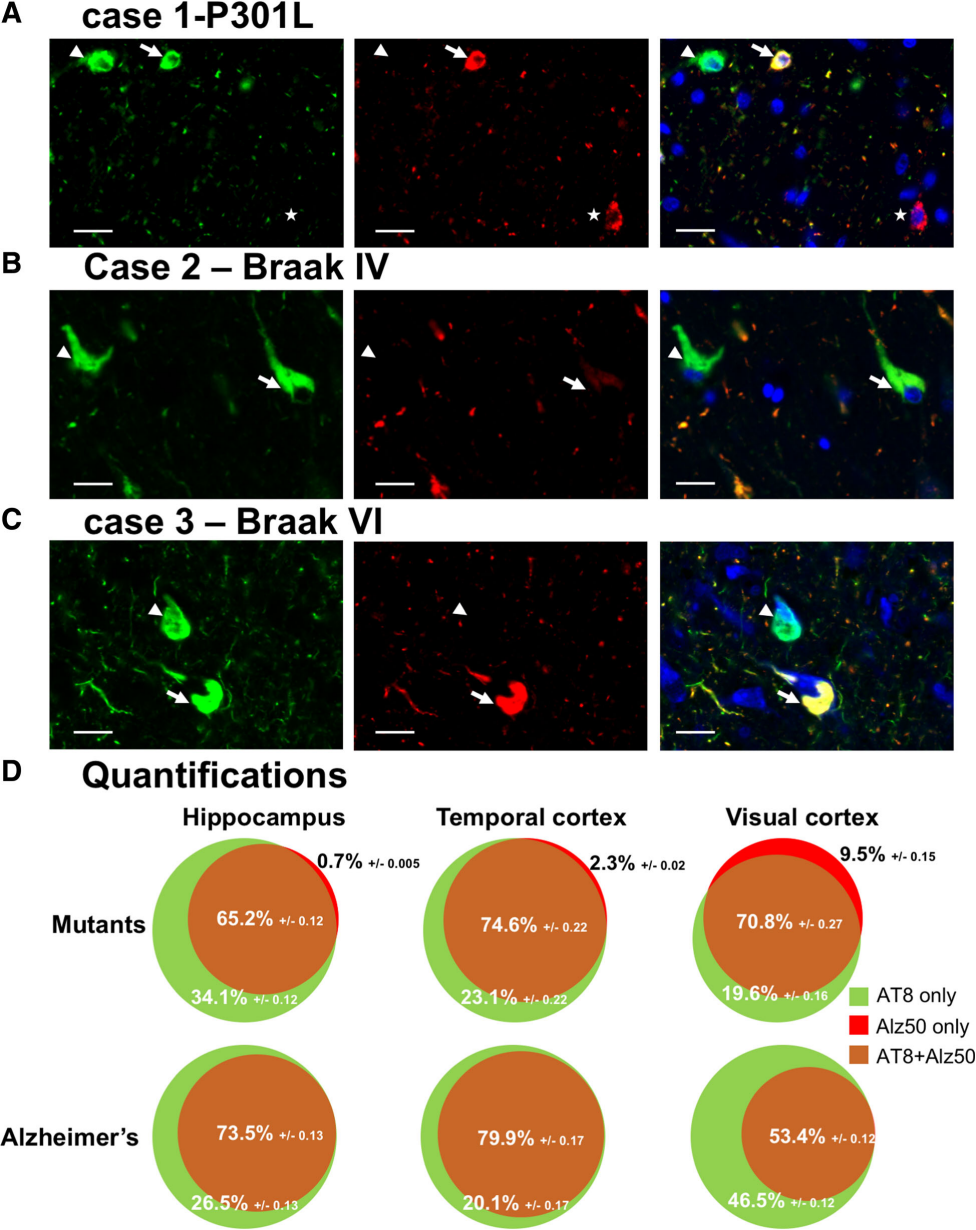
Tau misfolding and hyperphosphorylation in human brains with AD and genetic FTLD-Tau. (**a, b** and **c**) human brain sections from a genetic FTLD-Tau case (**a**), a Braak IV AD case (**b**) and a Braak VI AD case (**c**) stained with AT8 (green), Alz50 (red) and Dapi (blue) showing neurons Alz50 and AT8 positive (arrows), neurons only AT8 positive (arrowhead) and neurons only Alz50 positive (star). Scale bars represent 20 μm (**d**) Quantification of the percentage of neurons single or double positive for Alz50 and AT8 in MAPT mutants (*n* = 4, top panels) or AD cases (*n* = 6, low panels) in hippocampus (left), temporal cortex (middle) and visual cortex (right). The percentages for each category: double positive (brown), AT8 only (green) and Alz50 only (red) are indicated along with standard deviations. Statistical test used: Pearson’s Chi-squared test with Yates’ continuity correction was used to assess the distribution of Alz50-only neurons and AT8-only neurons in mutant versus AD cases. The presence of Alz50-only positive neurons was significantly linked to the presence of a MAPT mutation both taking into account all regions (*p* < .001; chi^2^ = 391) and in the hippocampus (*p* < .001; chi^2^ = 656). The presence of AT8-only positive neurons could only be linked with the presence of a mutation taking into account all regions (*p* < .001; chi^2^ = 171)

We hypothesized that the mechanisms of tau deposition are different in sporadic tauopathies than when a mutation of MAPT gene is present. Therefore, we investigated the presence of tau misfolding and hyperphosphorylation epitopes in human brain samples. We selected six Alzheimer’s disease patients (two Braak I, two Braak IV, two Braak VI) and 4 patients with Fronto-temporal dementia associated with a mutation of MAPT gene (two P301L, one P332S and one G389R). We stained brain sections from three different regions following the Braak stages: hippocampus, temporal cortex and visual cortex with AT8 antibody for tau hyperphosphorylation and Alz50 for tau misfolding. Three different phenotypes can be observed: neurons positive for both Alz50 and AT8 (Fig. 1a-c, arrows), neurons positive only for AT8 (Fig. 1a-c, arrowheads) and, more rarely, neurons positive only for Alz50 (Fig. 1a, star). We quantified in each case the number of neurons in each category. We counted a total number of 27,214 neurons for the AD cases and 15,460 for the mutant cases. Most of the neurons were positive both for tau misfolding and hyperphosphorylation in all cases (AD: *n* = 17,588, mutants *n* = 12,516 Fig. 1d, Additional file 1: Figure S1 and Additional file 2: Table S1). The number of neurons with only tau hyperphosphorylation was also prominent most of the time (AD: n = 17,588, mutants *n* = 2731 Fig. 1d, Additional file 1: Figure S1 and Additional file 2: Table S1). More interestingly, we found significantly more Alz50 positive-only neurons (213) in mutants compared to only 4 in AD cases (only in the visual cortex of Braak VI cases, Fig. 1d, Additional file 1: Figure S1 and Additional file 2: Table S1). Distributions of frequencies of both Alz50-only or AT8-only neurons were compared in mutant versus AD using a Pearson’s Chi-squared test with Yates’ continuity correction both globally (all regions pooled) and in the hippocampus. Interestingly, and taking into account the limitations of statistical analysis on such heterogenous cohort with a small number of cases, there is a significant link between having a mutation and having Alz50-only positive neurons both overall (*p* < .001; chi^2^ = 391) and in the hippocampus (p < .001; chi^2^ = 656). There is also a significant link between having a mutation and having AT8-only positive neurons overall (p < .001; chi^2^ = 171) but not in the hippocampus (*p* = 0.43; chi^2^ = .64). These results suggest that in AD, hyperphosphorylation precedes or accompanies misfolding in a large majority of neurons. By contrast, when the MAPT gene is mutated, misfolding seems to precede hyperphosphorylation in a small portion of neurons arguing for folding differences of mutant tau proteins.

### Cell-to-cell transfer of all tau species

**Fig. 2:**
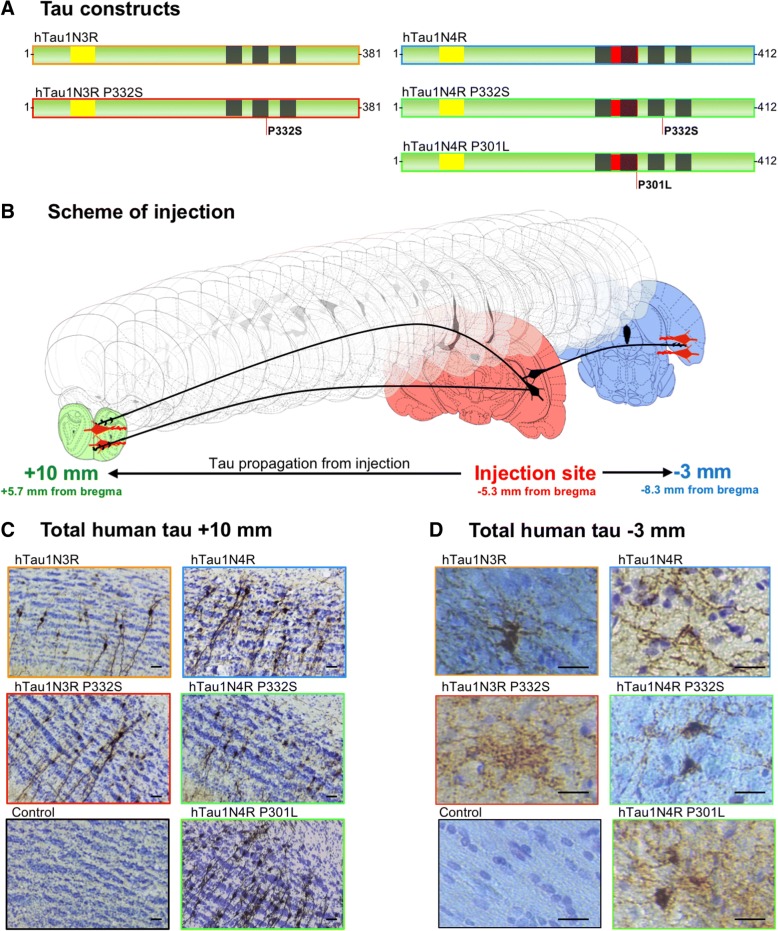
All tau species are transferred from neuron-to-neuron. **(a)** Schematic representations of tau construct used in this study. **(b)** Schematic representation of rat brain showing the injection coordinates in red and some of the rostral-most and caudal-most regions where we can find tau propagation. **(c** and **d)** Rat brains sections at coordinates + 5.7 mm **(c)** -8.3 mm **(d)** from bregma stained with a total human tau antibody (Adx215) showing transfer of tau for every species. htau1N4R (*n* = 9), htau1N4R-P301L (*n* = 5), htau1N4R-P332S (n = 6), htau1N3R (*n* = 8) or htau1N3R-P332S (*n* = 5). Scale bars represent 20 μm

We observe in human brains that hyperphosphorylation seems to appear first in sporadic cases such as AD patients but appears after misfolding in genetic FTLD-Tau. We next tested this hypothesis in an animal model. We described, in a previous study, the transfer of human tau proteins from the rat hippocampus to different distant secondary regions including limbic or olfactive regions following the injection of LVs encoding human wild-type 4R-tau [19]. Using this model of tau propagation, we wanted to assess whether different tau species (mutant tau and 3R-tau) act in a similar manner and also propagates from neuron-to-neuron. Different cohorts of Wistar male rats were bilaterally injected into the CA1 layer of the hippocampus with LVs encoding the human 3R-tau or 4R-tau either mutant or WT. We selected two different mutations: the widely used P301L only present on 4R-tau isoforms and the mutation P332S present on all isoforms [16] resulting in 5 different groups of animals referred above as 3R-tau, P332S-3R-tau, 4R-tau, P301L-4R-tau and P332S-4R-tau (Fig. 2a). We stained by immunohistochemistry the brain sections with a human specific N-terminal tau antibody (ADx215) in order to properly discriminate the exogenous over-expressed tau from the endogenous tau. With similar level of expression (Additional file 3: Figure S2) and no observable retrograde transfer of the viral vectors [19], 8 months post-injection, tau proteins can be detected in cell bodies in various long-distance regions such as limbic areas, cortical areas or olfactory area for all the tested groups of animals (Figs. 2b-c). This transfer is particularly observable in the granular cell layer of the olfactory bulb more than 10 mm away from the injection site (Fig. 2c) and in cortical caudal regions situated 3 mm behind the injection (Fig. 2d). These data clearly indicate that all species of tau, regardless of being 3R or 4R-tau, mutants or WT, transfer from neuron-to-neuron.

### Tau species-dependent differential propagation of tau pathology

We previously highlighted major differences between WT 4R-tau and P301L-4R-tau regarding hyperphosphorylation, misfolding and aggregation in this rat model [19]. The data we present above on human brains also argue for a difference between WT and mutant tau in the folding properties.

**Fig. 3:**
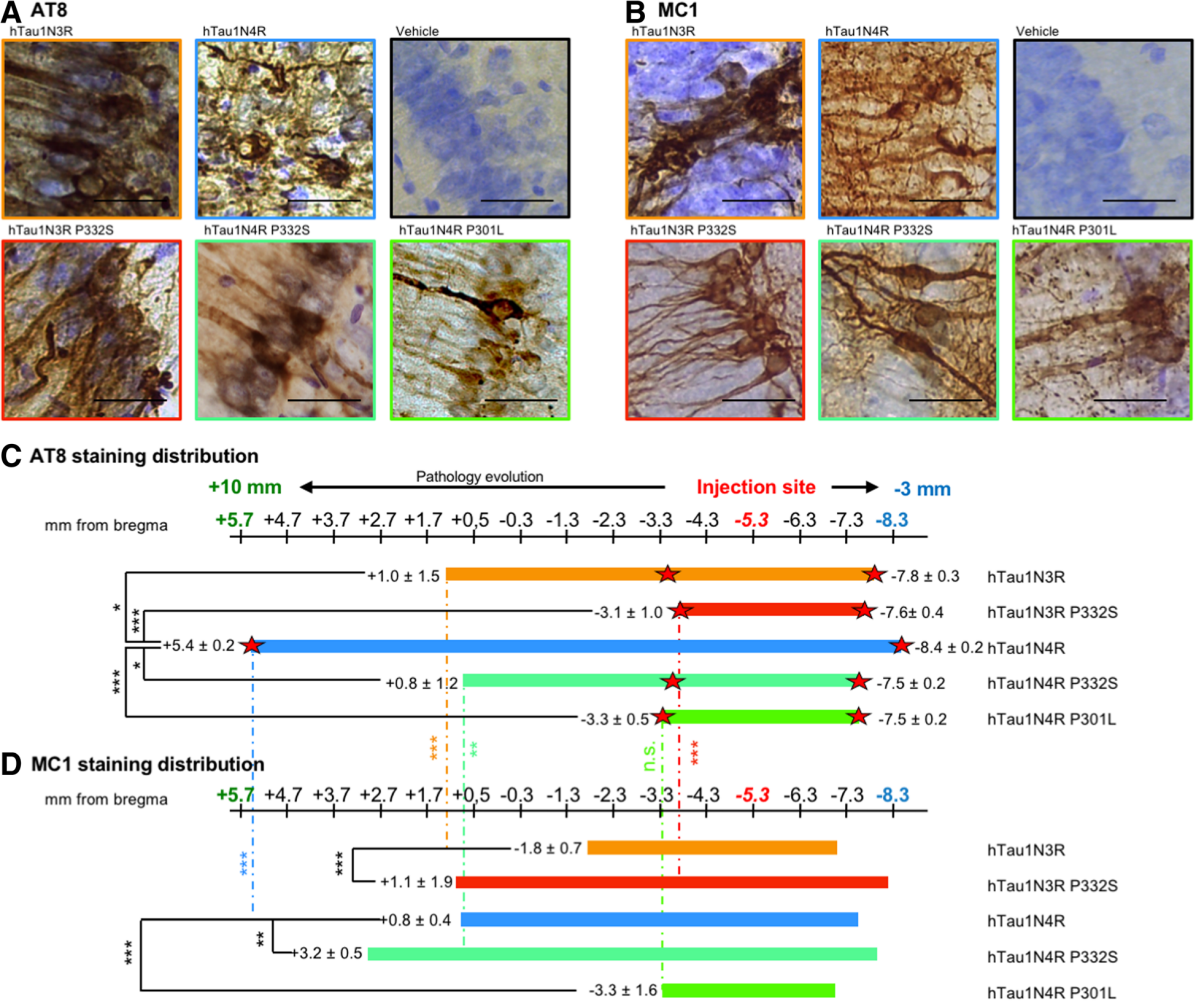
Differential of spreading of tau pathology between tau species. **(a** and **b)** Rat brains sections at − 5.3 mm from bregma for each group labelled with AT8-hyperphosphorylated tau antibody **(a)** or MC1-misfolded tau antibody **(b)** and showing strong tau pathology at the injection site. Scale bars represent 40 μm **(c** and **d)** Distribution of the AT8 **(c)** or MC1 **(d)** staining in coronal sections throughout the brains for each group is represented with bars along the scale of antero-posterior coordinates from Bregma. Bars represents the average of rostral-most to caudal-most staining (cell body or neurite) observed for each cohort. Red stars represent the coordinate of the last cell body observed. htau1N4R (*n* = 9), htau1N4R-P301L (*n* = 5), htau1N4R-P332S (*n* = 6), htau1N3R (*n* = 8) or htau1N3R-P332S (*n* = 5). The mean and standard deviations are indicated at the end of each bars. Statistical test used: One-way ANOVA test followed by a Tuckey post-hoc test was used to assess statistical differences between each group

Based on these data, we investigated the impact of tau isoforms and mutations on tau pathology spreading. We stained the brain sections with epitopes of tau pathology: hyperphosphoryled tau using the antibody AT8 [42, 44, 53] and misfolded tau using the antibody MC1 (IgG version of Alz50 antibody, [34, 36]). We first verified that, at the injection site, the focal overexpression of both constructs resulted in the formation of tau pathology in the CA1 of rats. 8 months post-injection, the neuronal expression of all isoforms, either mutant or WT, results in the formation of strong tau hyperphosphorylation (Fig. 3a) and misfolding (Fig. 3b).

In order to understand how tau pathology spreads through the brain, we analyzed the presence of tau lesions in the whole rat brain, from the olfactory bulb (rostro-caudal coordinates + 6 mm from bregma in coronal sections) to the end of the cortical areas (rostro-caudal coordinates − 9 mm from bregma in coronal sections). As a sensor for tau pathology spreading from the initiation site to secondary regions, we determined for each animal the rostral-most and caudal-most coordinates where we could see tau pathology, either in neurites or in cell bodies. We confirmed our previous data showing that in rats injected with LVs encoding 4R-tau the AT8 immunoreactivity was found in the cell body of second order neurons in several connected regions (e.g. the granular layer of the olfactory bulb) as far as 11 mm away from the injection site. Interestingly, in rats injected with LVs encoding P332S-4R-tau, P301L-4R-tau, 3R-tau or P332S-3R-tau, AT8 immunoreactivity was significantly restricted to the vicinity of the initiation site both for neurites or cell bodies (Fig. 3c).

In order to compare the appearance of tau hyperphosphorylation and tau misfolding in our model, we also stained by immunohistochemistry the epitope of misfolding MC1 in the different cohorts of rats. We determined the rostral-most and caudal-most coordinates for each animal to be able to compare the spatial appearance of MC1 epitope and of AT8 epitope. In the animals overexpressing the WT proteins 3R-tau and 4R-tau, the MC1 immunolabelling stayed significantly closer to the initiation site than the AT8 immunolabelling (Fig. 3d). Indeed, numerous brain regions show hyperphosphorylated tau without misfolding tau. It is really intriguing to notice that when a mutant tau species is overexpressed, the MC1 immunolabelling is retrieved in regions significantly more (or equally for P301L) distant from the initiation site than the AT8 immunolabelling (Fig. 3d) clearly suggesting a difference between WT proteins and mutant proteins in terms of tau misfolding and hyperphosphorylation in the context of spatial spreading.

## Discussion

The data in the present study reveal new aspects of the propagation of tau proteins by demonstrating that different species of tau have different behaviors in terms of pathological spreading and folding properties. Since the discovery of tau protein as the principal component of NFD [7, 26], around 20 pathologies involving aggregated tau were described with various time-courses, lesions and involved tau species [39]. This group includes pathologies with aggregated 3R-tau, 4R-tau, or both isoforms. Rare fronto-temporal dementia cases also involve mutation in the tau gene [23]. These latter proteins are pro-aggregative [4, 12] often showing strong tau pathology when overexpressed and therefore are widely used in the modelling of tau pathologies [18]. The in vivo studies dealing with tau propagation are also mainly based on the overexpression or injection of mutant 4R-tau proteins [1, 13, 14, 32, 41, 63]. However, in view of the neuroanatomical and biochemical differences among human tauopathies [2, 4, 22], one may ask if the different tau species trigger the same pathological mechanisms.

Here, we studied the misfolding and hyperphosphorylation status of tau proteins in human brains with AD or with MAPT mutations. In all groups, we found a majority of tangles showing both misfolding and hyperphosphorylation and also a high number of neurons showing only hyperphosphorylation. More interestingly though, the misfolding-only neurons were most prevalent in mutant tau brains (Fig. 1). This result doesn’t show per se that tau misfolding precedes hyperphosphorylation in MAPT-mutant patients and the contrary in AD patients but seems to indicate that the mutant-tau may have different folding properties compared to WT tau.

To model such differences, we took advantage of lentiviral technology to induce the accumulation of 3R or 4R WT or mutant tau, and to investigate the propagation of both the protein and the pathology. We show that regardless of the isoform or mutation, all tau proteins are capable of long-distance propagation through the brain (Fig. 2) consistent with the existence of a cell-to-cell transfer mechanism as previously suggested [14, 19, 41]. Our study indicates that most of the tau species, travel in a non-pathological, non-phosphorylated, non-misfolded state. Indeed, we clearly see transfer for every species studied (Fig. 2) but only some of them show long distance pathological epitopes presence (Fig. 3). It is clear in this model that at least part of tau cell-to-cell transfer is physiological, as tau does not seem to be either misfolded or hyperphosphorylated. This finding is in line with numerous studies showing that secreted tau is mostly monomeric and non-phosphorylated [11, 17, 37, 38, 47, 50–52, 56, 58, 62, 64, 70].

We also show differences in tau pathology between species of tau in the human brains (Fig. 1). Therefore, we wondered if, in the rat model, the propagation of pathological epitopes is impacted by the species. Indeed, all constructs trigger the development of tau pathology in the hippocampus of rats, but these pathologies evolve in a different manner in the whole brain. 4R-tau leads to a strong long-distance spreading of tau pathology when 3R-tau or mutant-tau mediated-pathologies stay in the vicinity of the pathology initiation site and don’t spread in long-distant brain areas (Fig. 3). These observations using mutant tau confirm our previous data obtained with the P301L mutation [19], located in the 2nd tau repeat, and extend our conclusions to another genetic FTLD-Tau mutation located in the 3rd tau repeat [16]. Given the reproducibility of our results among several cohorts of animals, as well as our observations in human brains, such differences between WT-4R-tau and 3R-tau or mutant-tau are likely to be due to intrinsic properties of mutant tau proteins. First, both mutant-tau and 3R-tau are known to induce better fibrillogenesis than WT-4R-tau [12, 59] probably due to conformational changes in the protein when tau protein is mutated [2, 20, 22]. For 3R-tau, the presence of a single cysteine in its sequence allows for the formation of intermolecular bridges initiating tau conversion/aggregation. Conversely, the two cysteines (C291, C322) present in 4R tau mostly drive the formation of intramolecular bridges, potentially slowing-down the process of oligomerization and subsequent aggregation [59].

Indeed, in this study, we suggest that 3R-, 4R- or mutant-tau support different types of pathological conversion. The classical view regarding the pathological conversion of tau proteins from a disordered state to insoluble, ordered and hyperphosphorylated aggregates suggests that tau becomes hyperphosphorylated inducing first the misfolding of the protein, and then its oligomerization. This hypothesis is further supported by the early appearance during the pathology of certain epitopes of phosphorylation [3, 43]. However, recently, Diamond’s team showed in a transgenic mouse model overexpressing the mutant P301S-tau that the seeding propensity of tau proteins is the first detectable indicator of tau pathology, before misfolding (MC1 antibody) and then hyperphosphorylation (AT8) [29]. We also previously reported the precocious appearance of tau misfolding epitopes and not hyperphosphorylation when mutant tau was overexpressed (As early as 2 months post lentiviral vectors injection, see [10]). By contrast, it is obvious that when overexpressing WT tau, hyperphosphorylation occurs first at the initiation site [10] but also in distant regions ([19], Fig. 3). Here, we confirm that when mutant-tau proteins accumulate, tau may first acquire misfolding properties (Figs. 1 and 3). This supports the existence of an intrinsic misfolding in mutant-tau proteins leading to the early appearance of a misfolding epitope and to the formation of fibrils with different structures [2, 20, 22]. Interestingly, the structure of these fibrils is transmissible to other tau species [20, 22]. This prominent misfolding might be the cause of the higher toxicity and neurodegeneration reported when mutant tau is expressed compared to WT [10, 30].

The phosphorylation state of tau may be a key player in the propagation processes. We know that tau is retained within axons due to its binding with microtubules which is highly dependent on phosphorylation [40]. Given that 3R-tau and mutant-tau show weaker binding to microtubules compared to 4R-WT-tau [9, 16, 27], it is very unlikely for 3R-phospho-tau and mutant-phospho-tau to stay in the axons. We hypothesize that they relocalize to the soma and therefore be less available for trans-synaptic transfer than 4R-tau. These results also suggest the presence of different tau species within the same individual (e.g. non-phosphorylated tau, phosphorylated tau, misfolded tau, truncated tau, dimers, oligomers, polymers, seeding-competent tau) that act differentially for tau transfer and pathological propagation. Most of them can be called “tau pathological species” but it is rather difficult to clearly identify the role of each in the pathology. In further studies, these different tau species should be analyzed independently to understand the part of each in the pathophysiological processes such as tau pathology spreading, misfolding or aggregation.

To conclude, the mechanisms of tau propagation and cell-to-cell transfer are highly dependent on tau species and our study is the first to identify this differential propensity of tau isoforms/mutations to mediate tau pathology spreading. This observation probably relies on intrinsic differences between tau species such as folding. These characteristics are consistent with what is observed in the human tauopathies and could explain their phenotypic specificities. This study also highlights the fundamental difference between tau physiological cell-to-cell transfer and tau pathological propagation. Those two mechanisms probably involve different species of tau that behave differently in the brain. This concept must be carefully taken into account and addressed in further studies dealing with tau propagation.

## Additional files

Additional file 1: **Figure S1.** Tau misfolding and hyperphosphorylation in human brains with AD and genetic FTLD-Tau-detailed figure. Details of each individual is indicated to show the patient-to-patient variability. Rows represent patients (numbered from 1 to 10), columns represent the regions studied. In each Venn diagram are indicated the percentage of neurons counted for each patient in each region. AT8 only neurons are indicated in green, Alz50-only in red and double-positive neurons in brown. MAPT mutants (*n* = 4), AD cases (*n* = 6). (TIFF 6255 kb)

Additional file 2: **Table S1.** Detailed neuronal counts for each patient are indicated here**.** MAPT mutants (*n* = 4), AD cases (*n* = 6). (DOCX 16 kb)

Additional file 3: **Figure S2.** No difference in transgene expression. Expression of MAPT gene in the different cohorts show no statistical difference between the expression of the different constructs. htau1N4R (*n* = 3), htau1N4R-P301L (*n* = 3), htau1N4R-P332S (*n* = 3), htau1N3R (*n* = 3) or htau1N3R-P332S (*n* = 3). Statistical test used: One-way ANOVA test followed by a Tuckey post-hoc test was used to assess statistical differences. (TIFF 5 mb)
