# Supplementary figures and images for: Different tau species lead to heterogeneous tau pathology propagation and misfolding
Source: Acta Neuropathol Commun. 2018 Nov 29;6:132. doi: 10.1186/s40478-018-0637-7 (PMC6263555; doi:10.1186/s40478-018-0637-7)

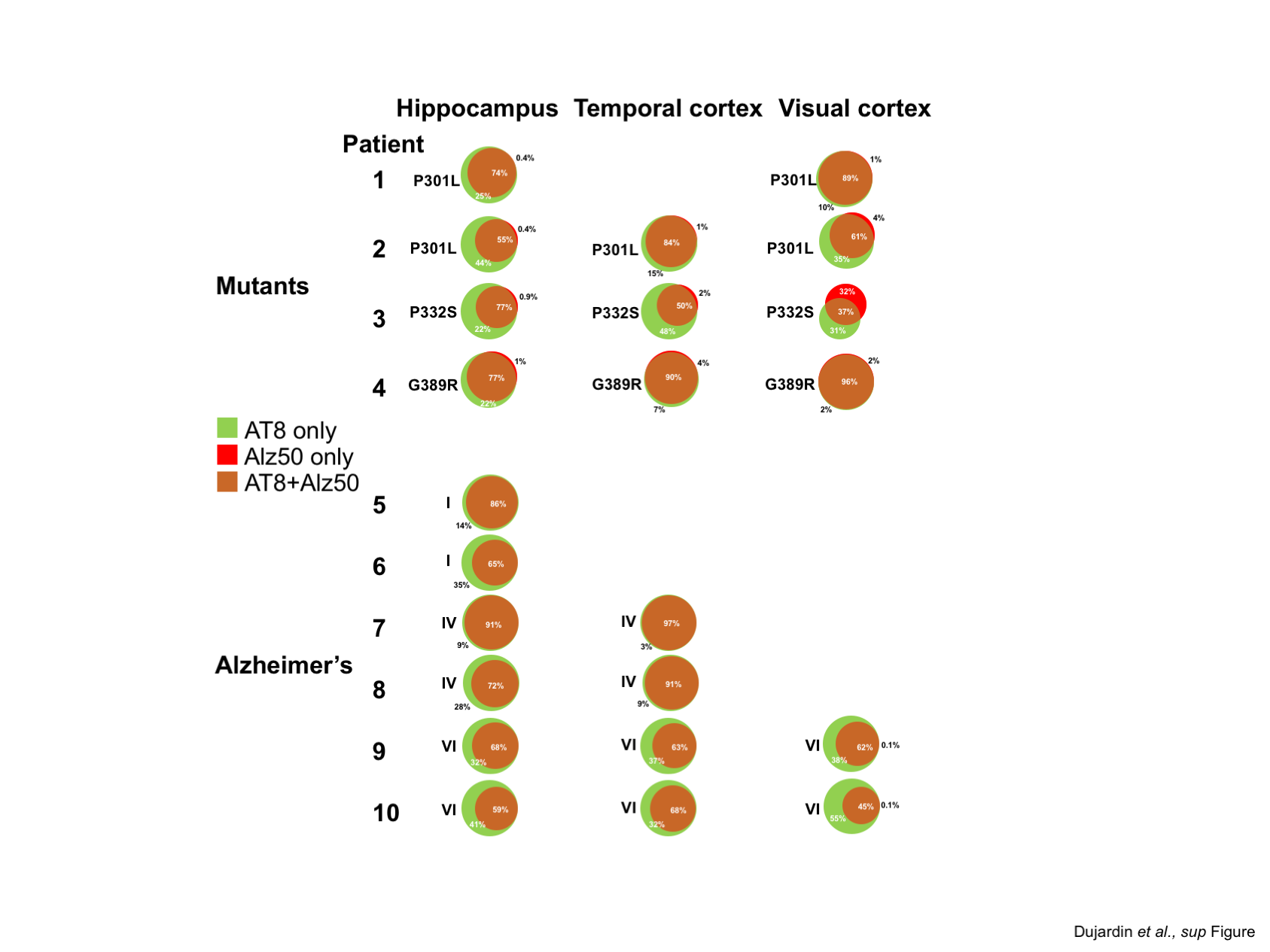

Supplement: Supplementary file 1 — Figure S1. Tau misfolding and hyperphosphorylation in human brains with AD and genetic FTLD-Tau-detailed figure. Details of each individual is indicated to show the patient-to-patient variability. Rows represent patients (numbered from 1 to 10), columns represent the regions studied. In each Venn diagram are indicated the percentage of neurons counted for each patient in each region. AT8 only neurons are indicated in green, Alz50-only in red and double-positive neurons in brown. MAPT mutants (n = 4), AD cases (n = 6). (TIFF 6255 kb) [file 40478_2018_637_MOESM1_ESM.tiff]

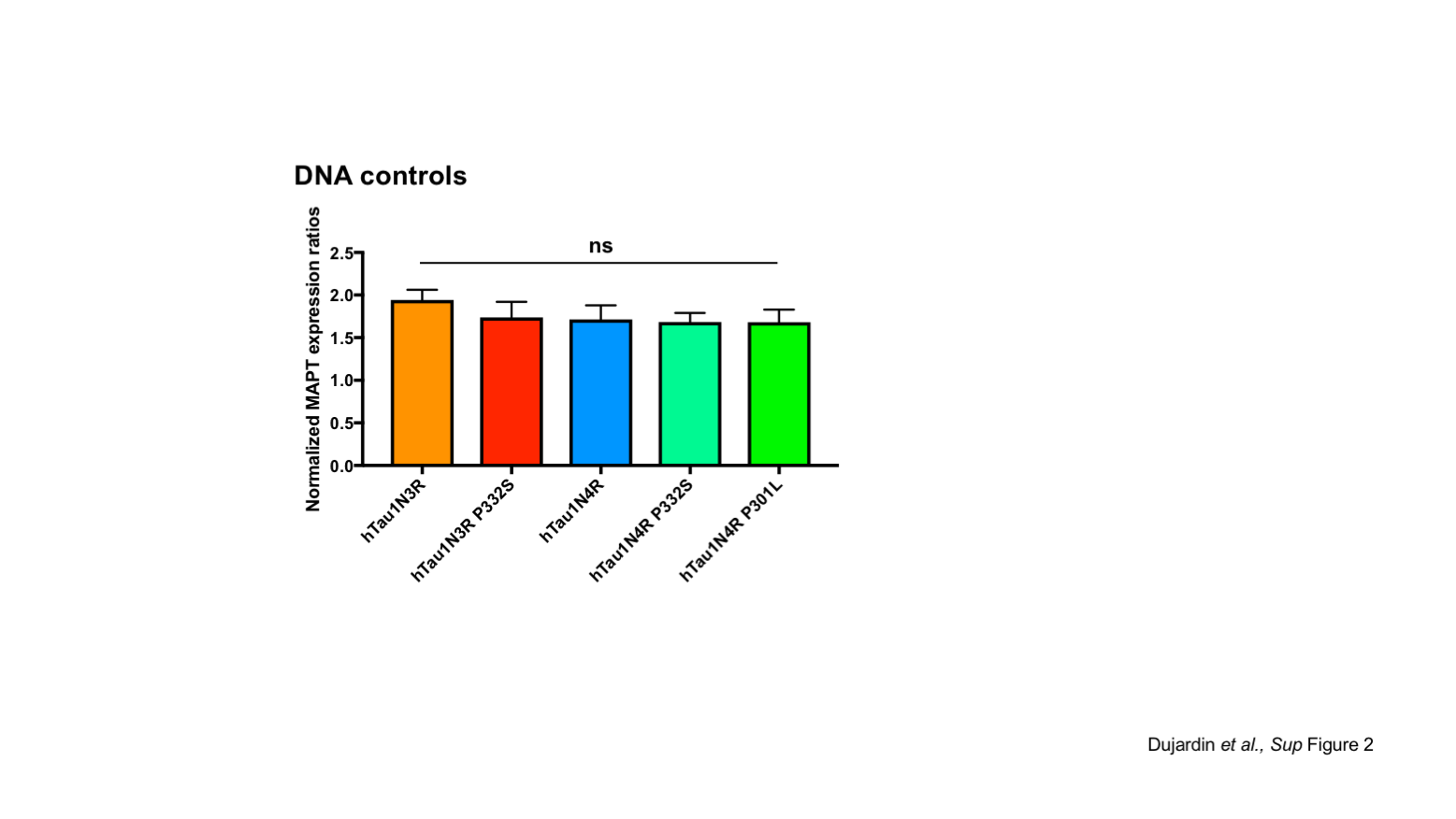

Supplement: Supplementary file 3 — Figure S2. No difference in transgene expression. Expression of MAPT gene in the different cohorts show no statistical difference between the expression of the different constructs. htau1N4R (n = 3), htau1N4R-P301L (n = 3), htau1N4R-P332S (n = 3), htau1N3R (n = 3) or htau1N3R-P332S (n = 3). Statistical test used: One-way ANOVA test followed by a Tuckey post-hoc test was used to assess statistical differences. (TIFF 5 mb) [file 40478_2018_637_MOESM3_ESM.tiff]
