# Supplementary material for: Different tau species lead to heterogeneous tau pathology propagation and misfolding
Source: Acta Neuropathol Commun. 2018 Nov 29;6:132. doi: 10.1186/s40478-018-0637-7 (PMC6263555; doi:10.1186/s40478-018-0637-7)
Supplement: Supplementary file 2 — Table S1. Detailed neuronal counts for each patient are indicated here. MAPT mutants (n = 4), AD cases (n = 6). (DOCX 16 kb) [file 40478_2018_637_MOESM2_ESM.docx]

|  |  |  | Hippocampus | | | Temporal Cortex | | | Visual Cortex | | |
| --- | --- | --- | --- | --- | --- | --- | --- | --- | --- | --- | --- |
| Case | Braak stage (if applicable) | MAPT mutation (if applicable) | AT8 only | Alz50 only | AT8 and Alz50 | AT8 only | Alz50 only | AT8 and Alz50 | AT8 only | Alz50 only | AT8 and Alz50 |
| 1 | N/A | P301L | 679 | 12 | 1977 | N/A | N/A | N/A | 488 | 30 | 4184 |
| 2 | N/A | P301L | 499 | 4 | 622 | 105 | 5 | 587 | 30 | 3 | 52 |
| 3 | N/A | P332S | 164 | 7 | 584 | 138 | 7 | 144 | 23 | 24 | 28 |
| 4 | N/A | G389R | 164 | 11 | 584 | 111 | 63 | 1509 | 52 | 47 | 2292 |
| 5 | I | N/A | 18 | 0 | 111 | N/A | N/A | N/A | N/A | N/A | N/A |
| 6 | I | N/A | 46 | 0 | 86 | N/A | N/A | N/A | N/A | N/A | N/A |
| 7 | IV | N/A | 155 | 0 | 1599 | 22 | 0 | 752 | N/A | N/A | N/A |
| 8 | IV | N/A | 456 | 0 | 1148 | 61 | 0 | 621 | N/A | N/A | N/A |
| 9 | VI | N/A | 779 | 0 | 1668 | 1758 | 0 | 3039 | 975 | 2 | 1590 |
| 10 | VI | N/A | 1516 | 0 | 2160 | 1443 | 0 | 3046 | 2293 | 2 | 1868 |
